# Girl child marriage, socioeconomic status, and undernutrition: evidence from 35 countries in Sub-Saharan Africa

**DOI:** 10.1186/s12916-019-1279-8

**Published:** 2019-03-08

**Authors:** Yvette Efevbera, Jacqueline Bhabha, Paul Farmer, Günther Fink

**Affiliations:** 1 Department of Global Health and Population, Harvard T. H. Chan School of Public Health, 665 Huntington Ave, Bldg. 1, 11th floor, Boston, MA 02115 USA; 2 FXB Center for Health and Human Rights, Harvard T. H. Chan School of Public Health, 651 Huntington Ave, 7th Floor, Boston, MA 02115 USA; 3 Department of Global Health and Social Medicine, Harvard Medical School, 641 Huntington Ave, Boston, MA 02115 USA; 4 Swiss Tropical and Public Health Institute and University of Basel, Socinstrasse 57, 4051 Basel, Switzerland

**Keywords:** Child marriage, Undernutrition, Women, Sub-Saharan Africa, Demographic and health surveys

## Abstract

**Background:**

Girl child marriage, a formal union of a female before age 18, and undernutrition remain common in Sub-Saharan Africa. The aim of this study is to establish the extent to which girl child marriage contributes to socioeconomic status and underweight, a measure of undernutrition, among adult women.

**Methods:**

We used data from 103 Demographic and Health Surveys (DHS), representing 35 African countries from 1991 to 2014. Girl child marriage was coded both as a binary variable (before 18 years) and categorical variable (before 14, 14 to 15 years, 16 to 17 years). The primary outcome was underweight (body mass index less than 18·5). Secondary outcomes were early and multiple childbearing, secondary education completion, and wealth index. Logistic regression models were used to estimate associations.

**Results:**

Fifty-five percent of women married before age 18. Girl child marriage was associated with reduced risk of being underweight both in models adjusted for basic confounders (risk difference = − 0.020, 95% CI [− 0.026, − 0.014], *p* < 0.01) and in models adjusted for childbearing, women’s relative status, and socioeconomic outcomes (risk difference = − 0.018, 95% CI [− 0.024, − 0.011], *p* < 0.01). Conditional on completing primary education and community fixed-effects, women married before 18 years had an increased risk of early motherhood (risk difference = 0.38, 95% CI [0.38, 0.38], *p* < 0.01) and of being in the poorest quintile (risk difference = 0.024, 95% CI [0.012, 0.036], *p* < 0.01), and were 27 percentage points less likely to complete secondary education (risk difference = − 0.27, 95% CI [− 0.28, − 0.26)], *p* < 001), compared to women married as adults.

**Conclusions:**

Though associated with substantially reduced socioeconomic status, girl child marriage appears to be associated with slightly reduced risk of being underweight in the population studied. Further research is needed to understand the determinants of undernutrition in this context as well as the broader relationship between socioeconomic status and nutritional outcomes.

**Electronic supplementary material:**

The online version of this article (10.1186/s12916-019-1279-8) contains supplementary material, which is available to authorized users.

## Background

Globally, over 700 million women alive today entered a formal union before age 18 [1]. In developing countries, one in nine girls marries before age 15 while one in three girls marries before age 18 [2]. Girl child marriage, defined as a female in a formal union before age 18, violates rights guaranteed in international and regional human right instruments and has been associated with adverse health behaviors and outcomes. Girl child marriage has been associated with increased fertility and reduced modern family planning, reduced antenatal care, and less safe delivery [3–5]. Literature has also documented significant associations between girl child marriage and mental health disorder diagnoses [6], suicide attempt and ideation [7], and items in measures of post-traumatic stress disorder, social reactions, abuse attributions, and self-esteem [8].

Though both ending child marriage and improving nutritional status are key items in achieving Sustainable Development Goals (SDGs) 2 and 5 by 2030, much less is known regarding empirical associations between girl child marriage and undernutrition [9, 10]. Globally, undernutrition is identified as the main cause for 3.5 million deaths in mothers and children and for 11% of disability-adjusted life years (DALYs) [11]. Among adolescent girls, being underweight, or too thin for age and height, ranges from 1 to 10% across Sub-Saharan Africa [12]. More than 10% of adult women are underweight in Sub-Saharan Africa, and despite improvement from 1980 to 1995, where the rate dropped from 18 to 11%, the proportion of women underweight is more than double to that of the Americas, Caribbean, and Europe [11]. Sub-Saharan Africa also has the highest proportion of countries with women married as children, as 18 of the 20 countries with the highest percentages of girl child marriage worldwide are found in the region [13]. If current trends in marriage and population growth continue, Sub-Saharan Africa will account for the largest number of girl child brides by 2050 [14].

**Fig. 1:**
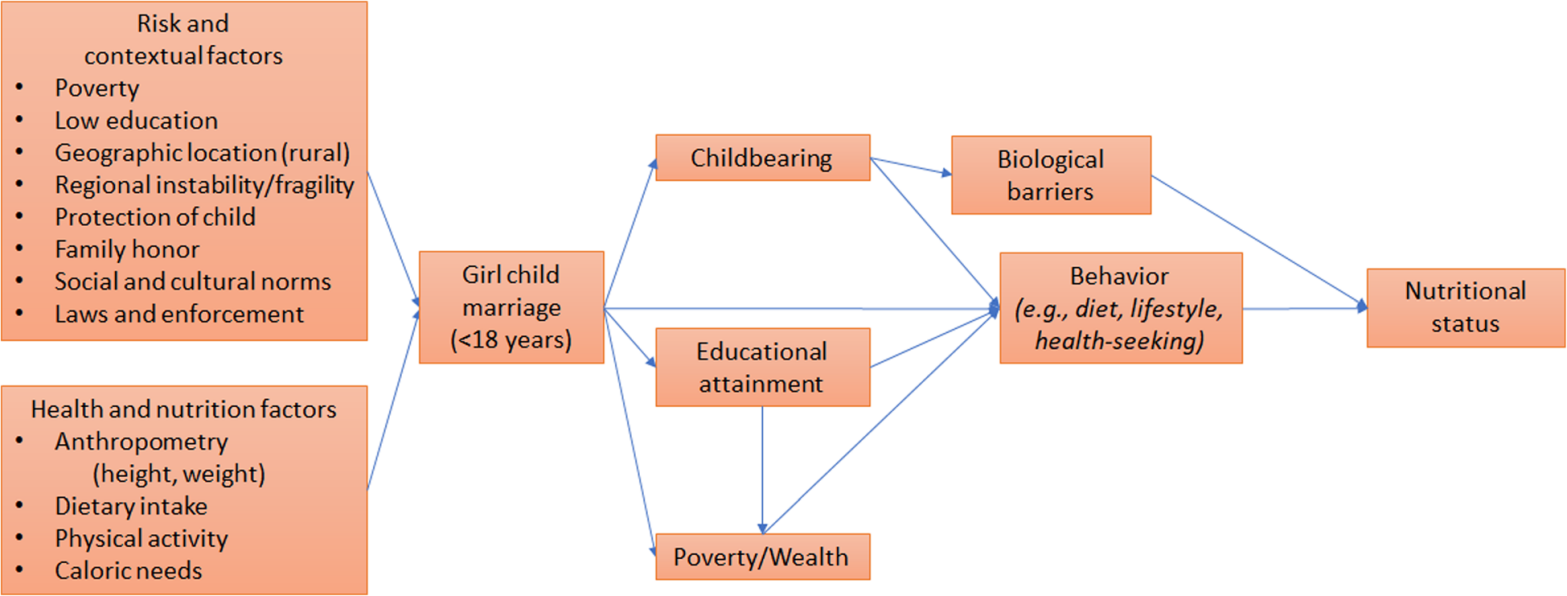
Conceptual model of how girl child marriage can impact health and nutritional status. Model was developed through extensive review of existing literature on girl child marriage, human development, and nutrition

The relationship between girl child marriage and undernutrition is not conceptually obvious. As illustrated in Fig. 1, girl child marriage may influence nutritional status through direct and indirect pathways. Marrying at earlier ages is often associated with early and multiple childbearing [15], leading to biological consequences for women’s nutritional status. Yet, early pregnancy has been associated with both increased and decreased maternal weight in different contexts [16, 17], influenced by a number of previous childbirths, fat-storing patterns, and dietary intake; the hypothesis for nutritional depletion following adolescent pregnancy has not been consistently observed, revealing that the direction of the relationship between early childbearing and weight change is unclear. Girl child marriage may also affect nutrition through social pathways [18]. Marrying at earlier ages has been associated with lower educational attainment [19], leading to more limited labor opportunities and income [20], weakening women’s socioeconomic status, and reducing autonomy. Both lower socioeconomic status and reduced autonomy will affect women’s decisions surrounding diet composition, physical activity, and health-seeking behavior [9]. A transition to marriage, additionally, can directly influence women’s physical activity levels as they take on new social roles in their contexts [21]. Yet, it is unclear in what direction these subsequent changes in women’s behavior would affect their nutritional status. These mechanisms—early and multiple childbearing, lower socioeconomic status, and reduced autonomy—may further lead to adverse health and developmental outcomes for children born to women who marry early [15, 18].

Using data from 103 Demographic and Health Surveys (DHS) from 1991 to 2014, representing 35 out of 48 African countries, the aim of this study was to estimate associations between girl child marriage, adult socioeconomic status, and the likelihood of being underweight (body mass index less than 18.5), as a measure of undernutrition, among women in Sub-Saharan Africa.

## Methods

### Data source

This study used data from the Demographic and Health Surveys (DHS) program. Implemented since 1984 by a private company called ICF under funding of the United States Agency for International Development (USAID), DHS are cross-sectional household-based surveys administered by national statistical offices and designed to be representative at national, residence, and regional levels [22]. DHS typically use a stratified two-stage cluster sampling design, randomly sampling from clusters, or enumeration areas (EAs), followed by households within each EA [22]. All women aged 15 to 49 within selected households are invited to complete the Women’s Questionnaire. In this study, we focused on DHS that collected height and weight data for women in Sub-Saharan Africa.

### Participants

Given the focus on medium- to long-term consequences of girl child marriage, we restricted analyses to women ages 20 to 49. According to World Health Organization guidelines, nutritional status in adolescents (ages 10 to 19) is measured differently than adults and cannot be directly compared [23]. For example, while body mass index is used to measure underweight in adults, low weight-for-age is traditionally used to measure underweight in children; measures of nutritional status in adolescents additionally accounts for age, and it is not until 19 years old that these curves approach convergence with adult models [24, 25]. We, thus, excluded women less than 20 years of age. Women over 49 years are not interviewed in DHS due to the survey’s historical focus on fertility and reproductive health. We also excluded survey respondents who stated they were pregnant at the time of survey because DHS does not compute body mass index for women who are pregnant [23]. We further restricted the sample to ever-married women (currently or previously married), given interest in measuring associations between age at marriage and nutritional status. Full information for the exposure and outcomes of interest was also required for inclusion.

### Measures

#### Girl child marriage

Girl child marriage, the exposure of interest, was defined as a self-reported formal union before the age of 18, based on international human rights guidelines [26]. In DHS, women were asked if they were currently married or if they had ever been married. Women were then asked for the month and year they started living with their husband, or how old they were when they first started living with their husband if year could not be provided. We ran additional analyses using an alternative categorical variable, comparing women married at age 18 years and above (adult marriage) with three child marriage age groups—below age 14, 14 to 15 years, 16 to 17 years—to test for differences by early and very early marital ages.

#### Underweight

Underweight status was the primary outcome of interest for this study. The body mass index (BMI), or the ratio of weight (kg) to square height in meters (m^2^), is an objective measure of women’s nutritional status. Weight and height of women were collected in DHS by a trained measurer [27]. Underweight was defined as having a BMI of less than 18.5. We also created a binary variable for being severely underweight (BMI less than 16) for additional analyses.

#### Secondary outcomes

We analyzed several secondary outcomes including age at first birth, number of children born, completion of secondary education, and wealth index. Secondary education was defined as an educational attainment of secondary school or higher. Wealth index was defined using asset quintiles as proxies for household relative socioeconomic status within a country, and asset scores were computed using principal component analysis of six key household assets following the methodology outlined by Filmer and Pritchett [28]. We compared wealth quintiles (poorest, poorer, middle, and richer) to the richest wealth quintile.

#### Other covariates

To reduce confounding, we controlled for self-reported completion of primary education or higher and cluster fixed-effects in adjusted analyses. In the second set of empirical models, we also controlled for age at time of interview (using a 5-year age group), age gap (calculated by subtracting the woman’s age from her partner’s age to account for age differential), and education level gap (calculated by subtracting the woman’s highest categorical level of schooling from her partner’s) to further reduce confounding concerns. There were no concerns of high correlations between girl child marriage and all covariates; thus, we included all variables in our final model (Additional file 1: Table S1).

### Statistical analysis

To assess the associations between girl child marriage and being underweight, a series of multivariable logistic regression models were estimated. In all models, we included EA fixed-effects to control for local differences in infrastructure, norms, and labor market opportunities that are likely to be correlated with both outcomes and predictors and thus could potentially cause confounding biases. For our main analyses, we estimated the basic association between girl child marriage and underweight, conditional on EA fixed-effects, and primary education completion. We included primary education in the base model because based on existing literature, we expect females in African contexts would have had the opportunity to complete primary schooling prior to their marriage, although secondary schooling would likely overlap, for some, with marriage. We thus conceptualized primary education as a predetermined confounding factor influencing the timing of a girl’s marriage [19, 29] and secondary schooling as often disrupted by early marriage instead [30]. In the second set of models, we estimated the same associations conditional on early and multiple childbearing, women’s completion of secondary education, wealth index, and partner characteristics that may influence women’s autonomy and decision-making power. The estimated coefficients on girl child marriage in this second set of models should be interpreted as direct effects outside of the three mechanisms directly accounted for (i.e., girl child marriage effects not working through women’s relative bargaining power, childbearing, or socioeconomic variables). Not all covariates were applicable to all participants (e.g., age at first birth, age gap with partner); thus, we also included dummy variables for ever given birth and currently married at the time of the survey. These coefficients were not reported and were instead used to prevent dropping of censored data. The missing data for age at first birth and age gap with partner variables were imputed using the mean value. We estimated associations for the pooled data and by country as well as accounting for different age at marriage categories.

Subsequently, we estimated the associations between girl child marriage and secondary outcomes: early and multiple childbearing, educational attainment, and poverty. These variables, used to proxy socioeconomic status, were identified as the most likely mechanisms from girl child marriage to undernutrition through an extensive literature review, resulting in the conceptual framework represented in Fig. 1. Finally, we ran several sensitivity analyses and additional analyses for country-specific models to further examine findings. We estimated additional models with severely underweight as an alternative outcome to examine a more extreme measure of undernutrition. We ran analyses restricting the sample to women age 20 to 24 to examine results for younger generations in the sample. We also ran analyses restricting data to only the most recent wave of data collection from 2011 to 2014 to examine if results were consistent in recent years. We ran analyses controlling for women’s work status within the survey year in a sub-sample to explore the effect of one proxy for women’s autonomy. To check for measurement error (i.e., to test if minor error in age reporting, which could lead to girl child marriage misclassification, would change study results), we ran analyses excluding women married at ages 18 and 19 to see if associations were present when accounting for more extreme child and adult marriage age categories. We also measured associations between girl child marriage and being anemic to see if the same relationship held for another measure of undernutrition. Results are presented as risk differences, with 95% confidence intervals and *p* values. Huber’s cluster-robust standard errors, which assume that clusters are independent, were used to account for within-group correlation due to the complex survey design used in the DHS [31].

Since we include EA fixed-effects (EA-specific intercepts) in all models, our empirical models exclusively explore within-EA variation, comparing women married at different ages within given EAs. From each EA, the DHS samples approximately 20 women who live in close proximity, typically a village in rural areas and a neighborhood in urban areas. Our empirical estimates should thus be interpreted as relative outcome differences of women marrying earlier in a community compared to other women from the same community marrying later. Analyses were conducted using StataMP 15 software.

### Ethical approval

This study was determined exempt by the Harvard Longwood Medical Area Institutional Review Board. Permission to use DHS data was obtained from the ICF/DHS program.

## Results

**Fig. 2:**
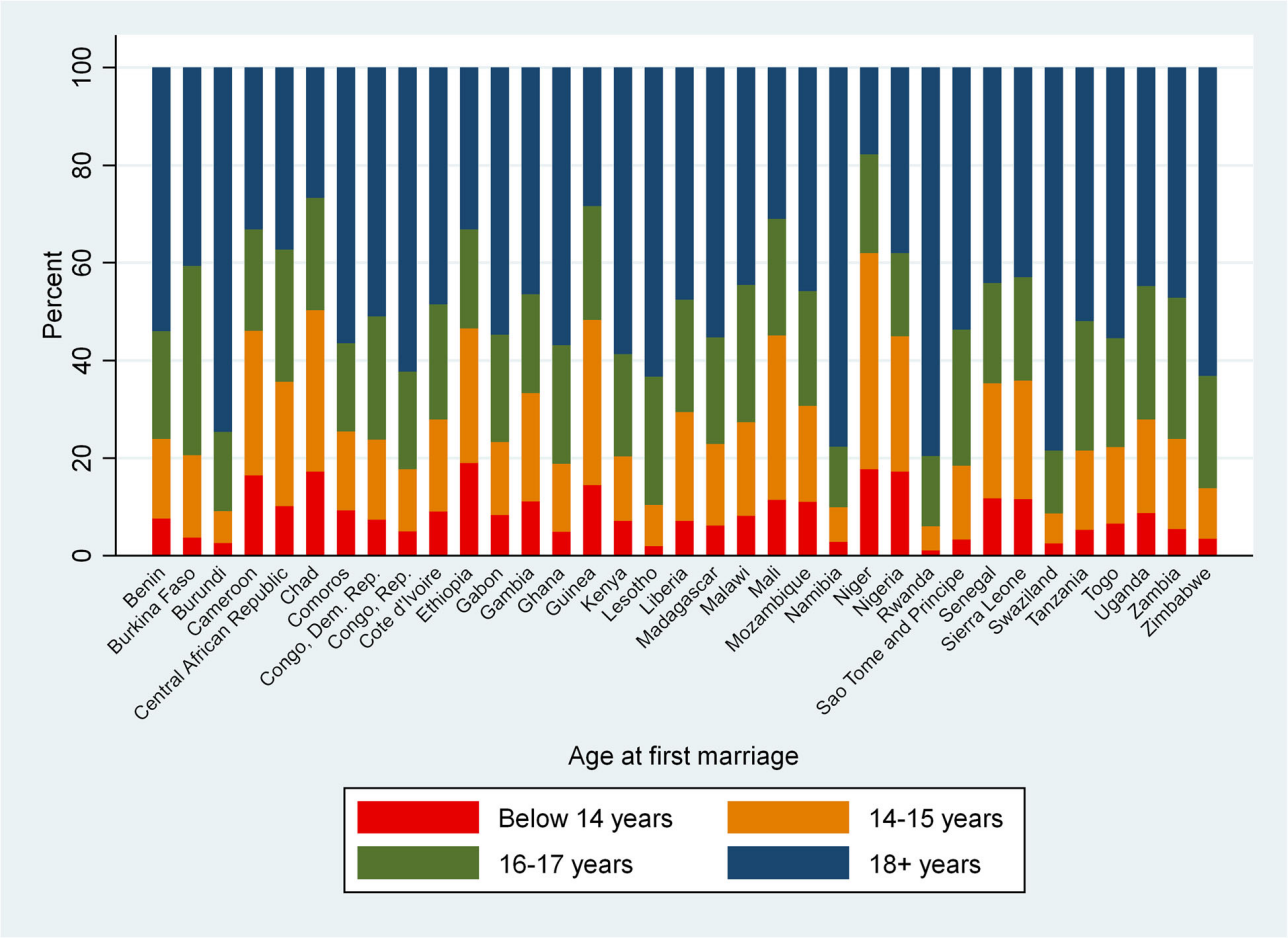
Percent of women by age at marriage and country among ever-married women age 20 to 49 included in final sample (*N* = 249,269)

The final sample included 249,269 women across 35 African countries (Additional file 2: Figure S2). Fifty-five percent of women married before age 18, with variation in marital age across the sample (Additional file 3: Figure S3). The percentage of women marrying before age 18 ranged from 19% in Namibia to 80% in Niger (Fig. 2). The median age at marriage for women who married as children was 15 years compared to 20 years for women who married as adults (Table 1). Overall, 18% of women in the sample were underweight while 2% were severely underweight; 22% of women were overweight, and 4% were obese (Additional file 4: Table S4). Sao Tome and Principe had the smallest proportion of women underweight (9%) while Ethiopia had the highest proportion (30%) (Additional file 5: Figure S5). Table 1 shows differences in the outcome and sociodemographic characteristics comparing women who married as adults and as children.

**Table 1:** Descriptive statistics of non-pregnant women aged 20 to 49 included in underweight analyses, by marital status (*N* = 249,269)

	Among adult marriage women (*N* = 111,724)	Percent	Among child marriage women (*N* = 137,545)	Percent
Exposure				
Age at marriage (median)	20		15	
Outcome				
BMI (median)	21.3		20.9	
Underweight	18,736	17	25,035	18
Covariate				
Age at interview (median)	32 years		31 years	
Currently married	97,291	87	122,630	89
Mother’s highest education level completed				
None	68,599	61.4	114,880	83.5
Primary	32,582	29.2	20,536	14.9
Secondary or higher	10,543	9.4	2129	1.6
Total number of births (median)	3		5	
Geographic location (urban)	34,111	31	29,217	21
Wealth quintile				
Poorest	38,020	34	55,521	40
Poorer	20,279	18	26,716	19
Middle	20,059	18	26,792	20
Richer	16,310	15	16,693	12
Richest	17,056	15	11,823	9
Partner age (median)	39		40	
Age gap with partner (median)	6		8	
Partner education				
None	58,264	52	97,091	71
Primary	35,186	32	30,305	22
Secondary or higher	18,274	16	10,149	7
Education gap (mean)	0.162		0.188	

*p* < 0.001 for difference between each variable in adult married and child married populations. Analyses clustered the standard errors at the country and survey cluster level

Table 2 shows the estimated risk differences for being underweight. Conditional on primary education and EA fixed-effects, women who married before age 18 had two percentage points lower probability of being underweight as compared to women who married at age 18 or above (risk difference = − 0.02, 95% CI [− 0.026, − 0.014], *p* < 0.01). The estimated association remained largely unchanged when we additionally adjusted for childbearing, women’s relative status, and socioeconomic outcomes (risk difference = − 0.018, 95% CI [− 0.024, − 0.011], *p* < 0.01). Additional file 6: Table S6 shows that similar results are observed when estimating associations among early and very early ages at marriage. Compared to women who married at age 18 or above, models adjusting for childbearing, women’s relative status, and socioeconomic status revealed that the probability of being underweight was two percentage points less for women married before age 14, just over two percentage points less for women married between ages 14 and 15, and three percentage points less for women married between 16 and 17 years. Results were not significant for the earliest marital age category by the final model.

**Table 2:** Risk difference of girl child marriage (binary) and underweight for pooled analysis (*N* = 249,269)

Variables	Model 1	Model 2
Girl child marriage (18+ years, ref.)	− 0.020** (− 0.026, − 0.014)	− 0.018** (− 0.024, − 0.011)
Completion of primary education (no, ref.)	−0.055** (− 0.064, − 0.047)	− 0.052** (− 0.060, − 0.043)
Current age (20–24 years, ref.)		
25–29 years		− 0.015** (− 0.023, − 0.0065)
30–34 years		− 0.014** (− 0.023, − 0.0043)
35–39 years		0.0016 (− 0.0093, 0.012)
40–44 years		0.014* (0.0016, 0.026)
45–49 years		0.034** (0.021, 0.047)
Age at first birth (years)		− 0.00072 (− 0.00164, 0.00020)
Number of children ever born		− 0.0086** (− 0.010, − 0.0071)
Completion of secondary education (no, ref.)		− 0.079** (− 0.096, − 0.061)
Wealth quintile (poorest, ref.)		
Poorer		− 0.014** (− 0.023, − 0.0061)
Middle		− 0.031** (− 0.039, − 0.022)
Richer		− 0.054** (− 0.064, − 0.044)
Richest		− 0.12** (− 0.14, − 0.11)
Age gap between partner and woman (years)		− 0.00043* (− 0.0010, − 0.000068)
Education gap between partner and woman (levels)		− 0.027** (− 0.033, − 0.022)

Coefficients presented are risk difference estimates from logistic regression models. Ninety-five percent CIs in parentheses are based on cluster standard errors. Underweight is defined as body mass index less than 18.5. Model 1 adjusts for sampling cluster and woman’s primary education. Model 2 adjusts for woman’s primary education, woman’s age, age at first birth, number of children born, secondary education, wealth quintile, and partner characteristics. Asterisks denote level of significance **p < 0.01, *p < 0.05

**Fig. 3:**
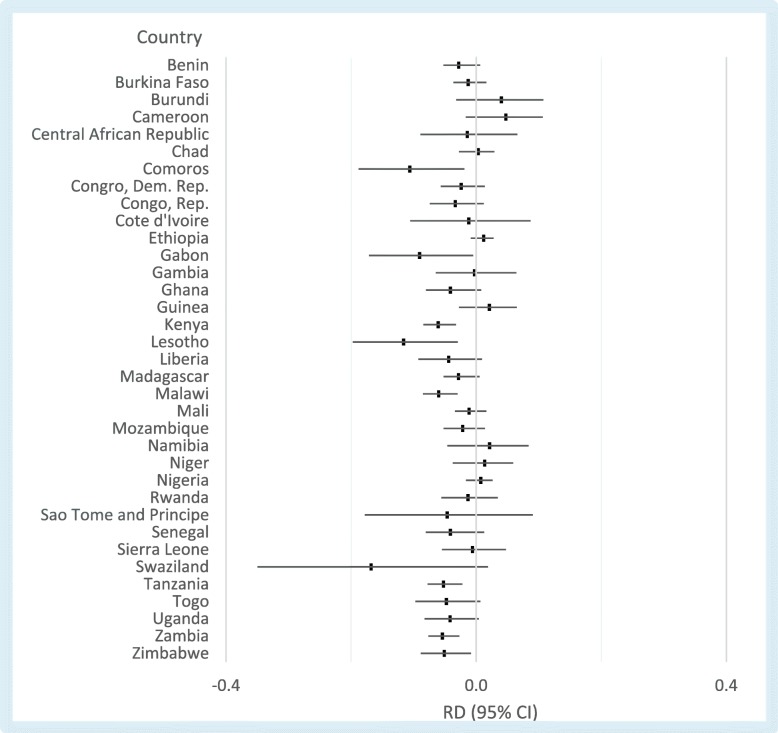
Country-specific associations between girl child marriage and female underweight. All models control for primary education and EA fixed-effects. Based on risk differences for 35 independent country-specific models

**Fig. 4:**
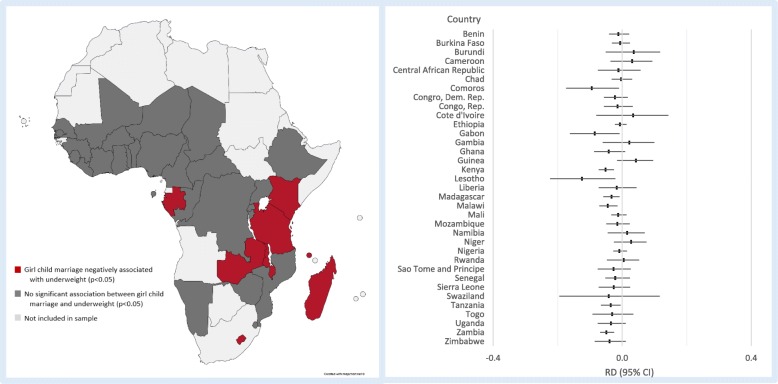
Map and country-specific associations between girl child marriage and underweight conditional on full set of covariates. Based on risk differences for 35 independent country-specific models controlling for primary education, age, age at first birth, number of children ever born, secondary education, wealth quintile, age gap, education gap, and EA fixed-effects

Figure 3 shows underweight risk differences for girl child marriage by country. Conditional on primary education and EA-fixed effects, eight countries across Central, Eastern, and Southern Africa showed marginally significant associations between girl child marriage and reduced risk of being underweight. Similar results were found for models adjusting for birth history, women’s relative status, and socioeconomic outcomes, with only Comoros, Gabon, Kenya, Lesotho, Madagascar, Malawi, Tanzania, and Zambia showing marginally significant and negative associations (Fig. 4). We found similar results when country-specific models were run using the categorical specification of girl child marriage (Additional file 7: Figure S7, Additional file 8: Table S8).

Table 3 shows associations between girl child marriage and secondary outcomes. Conditional on primary education and EA-fixed effects, women who married before age 18 had 38 percentage points increased probability of giving birth before age 18 (risk difference = 0.38, 95% CI [0.38, 0.38], *p* < 0.01) and 27 percentage points increased probability of having at least four children (risk difference = 0.27, 95% CI [0.26, 0.28], *p* < 0.01), compared to women who married as adults. Among women who completed primary school, girl child marriage was associated with a 27 percentage point reduction in the likelihood of completing secondary education compared to those who married as adults (risk difference = − 0.27, 95% CI [− 0.28, − 0.26], *p* < 0.01). Women who married before age 18 also had increased risk of being in the poorest quintile (risk difference = 0.024, 95% CI [0.012, 0.036], *p* < 0.01).

**Table 3:** Risk difference of girl child marriage and childbearing, secondary education, and household wealth

Panel 1: Age at first birth			
Variables			
Girl child marriage (18+ years, ref.)	Below 16 years old (16+ years, ref.)	Below 18 years old (18+ years, ref.)	
	0.34** (0.34, 0.34)	0.38** (0.38, 0.38)	
Panel 2: Number of children			
Variables			
Girl child marriage (18+ years, ref.)	1 child (none, ref.)	2 to 3 children (none, ref.)	4 or more children (none, ref.)
	0.078** (0.059, 0.096)	0.21** (0.20, 0.22)	0.27** (0.26, 0.28)
Panel 3: Secondary education			
Variables			
Girl child marriage (18+ years, ref.)	Completion of secondary education or higher (no, ref.)		
	− 0.27** (− 0.28, − 0.26)		
Panel 4: Wealth index			
Variables			
Girl child marriage (18+ years, ref.)	Poorest quintile (richest quintile, ref.)	Poorer quintile (richest quintile, ref.)	
	0.024** (0.012, 0.036)	0.050** (0.035, 0.066)	

Coefficients presented are risk difference estimates from logistic regression models estimating associations between girl child marriage and secondary outcomes, with 95% CIs in parentheses, controlling for EA fixed-effects and primary education and clustering standard errors at sample cluster level. For each secondary outcome, only women belonging to clusters in which there is variation in the outcome are included. Girl child marriage is defined as marriage before age 18. All variables are self-reported by participants. Secondary education estimates restrict to women who completed at least primary education. Wealth index was based on principal component analysis of household ownership of 6 core items (type of toilet facility, source of drinking water, main floor material, main wall material, and main roof material). **p < 0.01, *p < 0.05

Sensitivity analyses confirmed study results, displaying reduced risk of being underweight in very few countries and suggesting that associations were not overall significant at the country level (Additional file 9: Figure S9, Additional file 10: Figure S10, Additional file 11: Figure S11, Additional file 12: Figure S12, Additional file 13: Figure S13). Girl child marriage was significantly associated with reduced risk of being severely underweight in only one country, Democratic Republic of Congo; no countries showed significant associations with anemia. When restricting data to the youngest generation of participants (ages 20 to 24), only one country, Malawi, showed significant and negative associations. In the most recent wave of data collection (2011 to 2014), associations showed a significant and reduced risk of being underweight in four countries and increased risk of being underweight in one country. Restricting to more extreme ages of child and adult marriages also confirmed the main study results and revealed that our models may slightly underestimate risk by including women married at 18 and 19 years old (Additional file 14: Table S14).

## Discussion

### Interpretation

The aim of this study was to estimate the empirical associations between girl child marriage, adult socioeconomic status, and the likelihood of being underweight in Sub-Saharan Africa. While results suggest that women who married before age 18 had substantially increased risk of early and multiple childbearing, lower educational attainment, and living in poverty, analyses revealed that girl child marriage was associated with a slightly reduced risk of being underweight, with variation by country.

To our knowledge, this is the first study to explore associations between girl child marriage, socioeconomic status, and underweight status in Sub-Saharan Africa. While the socioeconomic associations found in this study highlight important negative long-term consequences of girl child marriage, the observed small, negative association with being underweight contradicts a recent study that found that the percentage of women underweight (thin) was significantly higher among those who married or gave birth before age 18 in two states of India [9]. While the authors of that study do not offer a direct explanation for their findings, they allude to roles of early childbearing, rural environments, illiteracy, and poverty in their discussion. These broad patterns are also visible in the DHS data used in this paper when women from different communities and districts are compared to each other (Additional file 15: Table S15). However, once we controlled for cluster-level confounding, using EA fixed-effects, these associations reversed, yielding the small, negative associations presented here. The pronounced difference between traditional cross-sectional estimates and models focusing on within-community comparisons only, like the ones presented here, suggests that the general cross-sectional relationship between girl child marriage and underweight is severely biased by local contextual factors that jointly determine marriage and nutritional outcomes.

Our findings additionally point toward differential experiences within marriage, particularly in the Sub-Saharan African context. An analysis of gender differentials of undernutrition in Sub-Saharan Africa found unique differences from South Asia and cautioned comparisons between the two contexts [32]. Svedeg (1990) posits that existing social customs including women’s participation in farming and marital practices such as early marriage, polygamy, and bride price paid by a groom’s family to his bride (rather than a dowry paid by the bride to a groom) are more common across Sub-Saharan Africa and are distinctly different than Asian contexts, possibly leading to more favorable nutritional status among women in Sub-Saharan Africa. These differences, moreover, may lead to a differential impact of the potential pathways outlined in Fig. 1 by context.

One possible explanation for our study results is that marriage provides an opportunity for a woman to have access to more food of different nutritional content. Women who marry earlier may more quickly access these nutritional outcomes and do not present as undernourished. Additionally, women who marry earlier, on average, give birth earlier, which has been associated with weight gain in some contexts [33]. Early and repeated pregnancies in adolescent mothers in a long-term prospective US study gained more weight than adult mother counterparts [16]. Similarly, weight change in childbearing and marriage may be associated with poorer nutritional status prior to marriage; a study in Pakistan found that women with increased levels of reproductive stress gained more weight and that those who were malnourished at baseline gained more than marginally nourished women [34]. In our analysis, the relationship between childbearing and underweight was rather weak overall. Further analysis of possible mediators of the association between girl child marriage and underweight may help to illuminate pathways.

Our results may also point toward the influence of contextual factors on marriage that further connect with a woman’s nutritional status, as identified in Fig. 1. For example, social and cultural norms in some African contexts point toward a preference for larger body size among women [35, 36], which may extend into bride preference. Evidence from developed countries further shows that married individuals are more likely to gain weight than unmarried counterparts, possibly explained by marital lifestyle changes [21, 37]. This study, thus, may capture the consequences of changing marital practices and family patterns in African contexts in an increasingly globalized world. Some participants from an ongoing qualitative study on child marriage in Guinea similarly describe how their early marriages, and the security provided, have supported good eating, sleeping, health, and access to healthcare [38]. As such, it would be important to look at associations between girl child marriage and overnutrition outcomes such as obesity, which has been positively associated with marriage in the US and is on the rise across the African continent. The double burden of malnutrition, another consequence of globalization, may mask negative nutritional and health outcomes in this study; we will explore these associations in a follow-up paper.

Beyond Sub-Saharan Africa, investigating the associations between girl child marriage and nutritional status would be important in any contexts where girl child marriage and undernutrition are prevalent, including South Asia, where this association has yet to be thoroughly explored. It is also important to note that the estimated associations between girl child marriage and undernutrition in our models—which adjust for the most likely mediators of childbearing, autonomy, and socioeconomic status—capture unobserved factors like nutrition intake, physical activity, or other unmeasured behavioral changes associated with girl child marriage. Additional research will strengthen an understanding on the potential consequences of girl child marriage, or the drivers of undernutrition, and could provide evidence for integrated or alternative programmatic and policy response.

### Limitations

This study used cross-sectional data and thus cannot claim causality. Though we control for several variables identified in the literature, residual confounding is possible. We were unable to control for other variables including pre-marital factors, such as anthropometry, diet and nutritional intake, and physical activity. Given that our data does not allow us to observe nutritional status prior to girl child marriage, it is possible that differential preferences for better-nourished brides or other contextual and sociocultural factors could be confounding factors in the observed relationships. We were also unable to control for childhood factors, such as early-life socioeconomic status and early-life nutrition, which likely impact women later in life. There may be variability in how data were collected across settings and timepoints. The analysis is also limited by our empirical approach which relies on EA fixed-effects and does not include DHS sampling weights. The main disadvantage of using EA fixed-effects is that only clusters with variation in both girl child marriage and underweight status are observed. The main disadvantage of not accounting for sampling weights is that results cannot be generalized to each country represented if the sample itself was not representative. Importantly, this study does not measure the short-term effects of nutritional changes immediately following childbirth and may, consequently, only capture long-term effects following adolescence. This study does not conduct mediation analysis but rather reports associations with secondary outcomes of interest; formal mediation analysis in this setting could be an important area for further research. Additionally, DHS data did not permit us to more thoroughly investigate country-specific sociocultural and historical contexts that may have contributed to marginally significant outcomes, presenting opportunities for future research.

### Strengths

Despite these limitations, this study has several strengths. We used a large dataset with nationally representative data from 35 African countries, greatly increasing generalizability and external validity compared to existing single-country or small sample studies on health consequences of girl child marriage. The outcome of underweight was calculated using measurements externally measured by trained individuals, rather than self-reported by participants, which could reduce bias. Data collection was standardized across countries and timepoints under the leadership of the DHS program, and in combination with the caution we exercised in pooling data, we are not overly concerned about data collection variability. Our analytical strategy using EA fixed-effects also addressed concerns of residual confounding, as we control for observed and unobserved differences within narrow geographic settings which may affect both girl child marriage and underweight status. By focusing on within-EA variation, we eliminated potential biases due to differences in observable and unobservable factors common to all women in these geographic areas, including local population density, infectious disease environment, poverty levels, availability of food and nutrients, differences in enforcement or presence of laws, cultural and social norms, fragility or effects of conflict, and other environmental factors.

## Conclusions

Girl child marriage remains a prevalent practice that threatens women’s socioeconomic status and rights more broadly. It continues to affect millions of women from adolescence, a time where rapid development takes place that has a lasting impact. Our findings suggest that girl child marriage is likely not a major driver of female underweight, emphasizing the importance of using empirical data to guide program and policy decision-making. Further research is needed to understand the determinants of undernutrition in this context as well as the broader relationship between socioeconomic status and nutritional outcomes.

## Additional files

Additional file 1: **Table S1.** Correlations between girl child marriage (binary) and other covariates. (DOCX 13 kb)

Additional file 2: **Figure S2.** Study flow chart on how sample size was determined. (DOCX 26 kb)

Additional file 3: **Figure S3.** Histogram of distribution of age at marriage among ever-married women age 20 to 49 included in final sample (*N* = 249,269) (DOCX 18 kb)

Additional file 4: **Table S4.** Prevalence of weight status (*N* = 249,269). *Note.* BMI refers to body mass index. (DOCX 13 kb)

Additional file 5: **Figure S5.** Scatter plot of mean age at marriage and proportion underweight by country of women age 20 to 49 included in final sample, with fitted line (*N* = 249,269). (DOCX 23 kb)

Additional file 6: **Table S6.** Risk difference of girl child marriage (categorical specification) and underweight for pooled analysis (*N* = 249,269). Note. Coefficients presented are risk difference estimates from logistic regression models. 95% CIs in parentheses are based on cluster standard errors. Underweight is defined as body mass index less than 18.5. Model 1 adjusts for sampling cluster and woman’s primary education. Model 2 adjusts for woman’s primary education, woman’s age, age at first birth, number of children born, secondary education, wealth quintile, and partner characteristics. Bolded values are significant at the *p* < 0.05 level. ***p < 0.01, **p < 0.05 (DOCX 16 kb)

Additional file 7: **Figure S7.** Country-specific associations by girl child marriage category and underweight. (DOCX 25 kb)

Additional file 8: **Table S8.** Sample sizes for country-specific sensitivity analyses. (DOCX 14 kb)

Additional file 9: **Figure S9.** Country-specific associations between girl child marriage (below 18 years) and severely underweight, conditional on full set of covariates. Note. All models control for primary education, age, age at first birth, number of children ever born, secondary education, wealth quintile, age gap, education gap, and EA fixed-effects. Based on 35 independent country-specific models. Two countries (Sao Tome and Principe and Swaziland) excluded due to lack of data or outcome variation by cluster. (DOCX 18 kb)

Additional file 10: **Figure S10.** Country-specific associations between girl child marriage (below 18 years) and being anemic (mild, moderate, or severe), conditional on full set of covariates. Note. All models control for primary education, age, age at first birth, number of children ever born, secondary education, wealth quintile, age gap, education gap, and EA fixed-effects. Based on 35 independent country-specific models. Seven countries excluded due to lack of data or outcome variation by cluster. (DOCX 18 kb)

Additional file 11: **Figure S11.** Country-specific associations between girl child marriage (below 18 years) and underweight for women aged 20 to 24, conditional on full set of covariates. Note. All models control for primary education, age, age at first birth, number of children ever born, secondary education, wealth quintile, age gap, education gap, and EA fixed-effects. Based on 35 independent country-specific models. One country (Swaziland) excluded due to lack of data and outcome variation by cluster. (DOCX 22 kb)

Additional file 12: **Figure S12.** Country-specific associations between girl child marriage (below 18 years) and underweight for 2011 to 2014 data, conditional on full set of covariates. Note. All models control for primary education, age, age at first birth, number of children ever born, secondary education, wealth quintile, age gap, education gap, and EA fixed-effects. Based on 35 independent country-specific models. Ten countries excluded due to lack of data or outcome variation by cluster. (DOCX 18 kb)

Additional file 13: **Figure S13.** Country-specific associations between girl child marriage (below 18 years) and underweight controlling for work status of woman, conditional on full set of covariates. Note. All models control for primary education, age, age at first birth, number of children ever born, secondary education, wealth quintile, age gap, education gap, and EA fixed-effects. We additionally control for whether or not the woman worked in the past year. Based on 35 independent country-specific models. (DOCX 18 kb)

Additional file 14: **Table S14.** Risk difference of girl child marriage (binary) and underweight for pooled analysis, excluding women married at age 18 or 19 (*N* = 184,828). Note. Coefficients presented are risk difference estimates from logistic regression models. Ninety-five percent CIs in parentheses are based on cluster standard errors. Underweight is defined as body mass index less than 18.5. Model 1 adjusts for sampling cluster and woman’s primary education. Model 2 adjusts for woman’s primary education, woman’s age, age at first birth, number of children born, secondary education, wealth quintile, and partner characteristics. Bolded values are significant at the *p* < 0.05 level. ***p < 0.01, **p < 0.05. (DOCX 16 kb)

Additional file 15: **Table S15.** Regression results of unadjusted associations between girl child marriage and underweight by country. Note. Coefficients presented are from unadjusted linear regression models with 95% CIs in parentheses. Underweight is defined as body mass index less than 18.5. ***p < 0.01, **p < 005. (DOCX 17 kb)

## Abbreviations

BMI: Body mass index
DALYs: Disability-adjusted life years
DHS: Demographic and Health Surveys
EAs: Enumeration areas
M: Model
SDGs: Sustainable Development Goals
USAID: United States Agency for International Development

## References

[1] Lendhardt A (2016). Every last girl.

[2] Loaiza SE, Wong S (2012). Marrying too young: end child marriage.

[3] Godha D, Hotchkiss DR, Gage AJ (2013). Association between child marriage and reproductive health outcomes and service utilization: a multi-country study from South Asia. J Adolesc Health.

[4] Nasrullah M, et al. Girl child marriage and its effect on fertility in Pakistan: findings from Pakistan demographic and health survey, 2006–2007. Matern Child Health J. 2013;18(3):534–43. 10.1007/s10995-013-1269-y 23580067

[5] Raj A (2009). Prevalence of child marriage and its effect on fertility and fertility-control outcomes of young women in India: a cross-sectional, observational study. Lancet.

[6] Le Strat Y, Dubertret C, Le Foll B (2011). Child marriage in the United States and its association with mental health in women. Pediatrics.

[7] Gage AJ (2012). Association of child marriage with suicidal thoughts and attempts among adolescent girls in Ethiopia. J Adolesc Health.

[8] Wondie Y (2011). Early marriage, rape, child prostitution, and related factors determining the psychosocial effects severity of child sexual abuse in Ethiopia. J Child Sex Abus.

[9] Goli S, Rammohan A, Singh D (2015). The effect of early marriages and early childbearing on women’s nutritional status in India. Matern Child Health J.

[10] Santhya K (2011). Early marriage and sexual and reproductive health vulnerabilities of young women: a synthesis of recent evidence from developing countries. Curr Opin Obstet Gynecol.

[11] Black RE (2013). Maternal and child undernutrition and overweight in low-income and middle-income countries. Lancet.

[12] Akseer N (2017). Global and regional trends in the nutritional status of young people: a critical and neglected age group. Ann N Y Acad Sci.

[13] UNICEF (2018). Percentage of women aged 20 to 24 years who were first married or in union before ages 15 and 18.

[14] UNICEF (2015). A profile of child marriage in Africa.

[15] Williamson, N., Motherhood in childhood: facing the challenge of adolescent pregnancy. The State of World Population 2013, in The State of World Population 2013, Robert W. Blum and Richard Kollodge, editors. 2013, United Nations Family Population Fund New York.

[16] Hediger ML, Scholl TO, Schall JI (1997). Implications of the Camden study of adolescent pregnancy: interactions among maternal growth, nutritional status, and body composition. Ann N Y Acad Sci.

[17] Rah JH (2008). Pregnancy and lactation hinder growth and nutritional status of adolescent girls in rural Bangladesh. J Nutr.

[18] Efevbera Y (2017). Girl child marriage as a risk factor for early childhood development and stunting. Soc Sci Med.

[19] Lloyd CB, Mensch BS (2008). Marriage and childbirth as factors in dropping out from school: an analysis of DHS data from Sub-Saharan Africa. Popul Stud (Camb).

[20] Filmer D, Fox L (2014). Youth employment in Sub-Saharan Africa. Africa development series.

[21] Eng PM (2005). Effects of marital transitions on changes in dietary and other health behaviours in US male health professionals. J Epidemiol Community Health.

[22] MeasureDHS/ICF International, Sampling and household listing manual, in Demographic and Health Surveys. 2012, United States Agency for International Development Calverton.

[23] WHO Expert Committee on Physical Status (1995). Physical status: the use and interpretation of anthropometry. WHO Techincal Report Series.

[24] Corsi DJ, Subramanyam MA, Subramanian SV (2011). Commentary: measuring nutritional status of children. Int J Epidemiol.

[25] de Onis M (2007). Development of a WHO growth reference for school-aged children and adolescents. Bull World Health Organ.

[26] The African charter on the rights and welfare of the child. Addis Ababa: Organization of African Unity (OAU); 1990.

[27] ICF International (2012). MEASURE DHS biomarker field manual.

[28] Rutstein SO, Johnson K (2004). The DHS wealth index. DHS comparative reports no. 6.

[29] Wodon Q, Nguyen MC, Tsimpo C (2016). Child marriage, education, and agency in Uganda. Fem Econ.

[30] Efevbera Y. ‘They did not give me the chance to finish my studies.’ The effects of child marriage on education in Guinea. In: Black scholarship matters: intellectualism, race, and the public sphere. Atlanta, Georgia: Black Doctoral Network; 2017.

[31] Huber PJ (1967). The behavior of maximum likelihood estimates under nonstandard conditions. Proceedings of the fifth Berkeley symposium on mathematical statistics and probability.

[32] Svedberg P (1990). Undernutrition in Sub-Saharan Africa: is there a gender bias?. J Dev Stud.

[33] Scholl TO, Hediger ML (1993). A review of the epidemiology of nutrition and adolescent pregnancy: maternal growth during pregnancy and its effect on the fetus. J Am Coll Nutr.

[34] Winkvist A (1994). Maternal energy depletion is buffered among malnourished women in Punjab, Pakistan. J Nutr.

[35] Ettarh R (2013). Overweight, obesity, and perception of body image mong slum residents in Nairobi, Kenya, 2008–2009. Prev Chronic Dis.

[36] Tuoyire DA, et al. Perceived ideal body size of Ghanaian women: “Not too skinny, but not too fat”. Women & Health. 2017;58(5):583–97. 10.1080/03630242.2017.1321607 28426342

[37] Sobal J, Rauschenbach B, Frongillo EA (2003). Marital status changes and body weight changes: a US longitudinal analysis. Soc Sci Med.

[38] Efevbera Y. Experiences of early and forced marriage in Conakry, Guinea: a grounded theory study. In: Society for adolescent health and medicine annual meeting: cultivating connections. New Orleans; 2017.

