# Supplementary material for: Girl child marriage, socioeconomic status, and undernutrition: evidence from 35 countries in Sub-Saharan Africa
Source: BMC Med. 2019 Mar 8;17:55. doi: 10.1186/s12916-019-1279-8 (PMC6407221; doi:10.1186/s12916-019-1279-8)
Supplement: Supplementary file 1 — Table S1. Correlations between girl child marriage (binary) and other covariates. (DOCX 13 kb) [file 12916_2019_1279_MOESM1_ESM.docx]

**Additional file 1: Table S1**

|  | coefficient | p-value |
| --- | --- | --- |
| Completion of primary education (no, ref.) | -0·2496 | p<0·001 |
| Current age (years) | -0·0371 | p<0·001 |
| Age at first birth (years) | -0·5432 | p<0·001 |
| Number of children ever born | 0·2335 | p<0·001 |
| Completion of secondary education (no, ref.) | -0·1786 | p<0·001 |
| Wealth quintile | -0·1058 | p<0·001 |
| Age gap between partner and woman (years) | 0·1386 | p<0·001 |
| Education gap between partner and woman (levels) | 0·0224 | p<0·001 |
| **Correlations between girl child marriage (binary) and other covariates** | | |
