# Supplementary material for: Girl child marriage, socioeconomic status, and undernutrition: evidence from 35 countries in Sub-Saharan Africa
Source: BMC Med. 2019 Mar 8;17:55. doi: 10.1186/s12916-019-1279-8 (PMC6407221; doi:10.1186/s12916-019-1279-8)
Supplement: Supplementary file 2 — Figure S2. Study flow chart on how sample size was determined. (DOCX 26 kb) [file 12916_2019_1279_MOESM2_ESM.docx]

**Additional file 2: Figure S2**

**Study flow chart on how sample size was determined**

711,964 women

aged 15 to 49

571,177 women

aged 20 to 49

Excluded:

140,787 women under age 20

508,473 non-pregnant women aged 20 to 49

Excluded:

61,909 women who were pregnant

795 women with missing pregnancy status

449,804 ever-married, non-pregnant women

aged 20 to 49

Excluded:

58,669 women who had never been married

Excluded:

751 women who had missing data on BMI

449,053 ever-married, non-pregnant women aged 20 to 49 with BMI data

298,217 ever-married, non-pregnant women aged 20 to 49 with BMI data in clusters with variation

Excluded:

150,836 women who belonged to clusters with no variation

Excluded:

48,948 women with missing data on covariates in final model

249,269 ever-married, non-pregnant women aged 20 to 49 with no missing data in clusters with variation
