# Supplementary material for: Girl child marriage, socioeconomic status, and undernutrition: evidence from 35 countries in Sub-Saharan Africa
Source: BMC Med. 2019 Mar 8;17:55. doi: 10.1186/s12916-019-1279-8 (PMC6407221; doi:10.1186/s12916-019-1279-8)
Supplement: Supplementary file 3 — Figure S3. Histogram of distribution of age at marriage among ever-married women age 20 to 49 included in final sample (N = 249,269) (DOCX 18 kb) [file 12916_2019_1279_MOESM3_ESM.docx]

Additional file 3: Fig. S3

**Histogram of distribution of age at marriage among ever-married women age 20 to 49 included in final sample (N=249,269)**
