# Supplementary material for: Girl child marriage, socioeconomic status, and undernutrition: evidence from 35 countries in Sub-Saharan Africa
Source: BMC Med. 2019 Mar 8;17:55. doi: 10.1186/s12916-019-1279-8 (PMC6407221; doi:10.1186/s12916-019-1279-8)
Supplement: Supplementary file 4 — Table S4. Prevalence of weight status (N = 249,269). Note. BMI refers to body mass index. (DOCX 13 kb) [file 12916_2019_1279_MOESM4_ESM.docx]

**Additional file 4: Table S4**

| **Weight status** | **n** | **%** |
| --- | --- | --- |
| Underweight (BMI<18.5) | 43,771 | 18 |
| Severely underweight (BMI<16) | 4,021 | 2 |
| Normal weight | 106,818 | 60 |
| Overweight (BMI=25+) | 98,680 | 22 |
| Obese (BMI=30+) | 10,726 | 4 |
| *Note.* BMI refers to body mass index. | | |

**Prevalence of weight status (N=249,269)**
