# Supplementary material for: Girl child marriage, socioeconomic status, and undernutrition: evidence from 35 countries in Sub-Saharan Africa
Source: BMC Med. 2019 Mar 8;17:55. doi: 10.1186/s12916-019-1279-8 (PMC6407221; doi:10.1186/s12916-019-1279-8)
Supplement: Supplementary file 5 — Figure S5. Scatter plot of mean age at marriage and proportion underweight by country of women age 20 to 49 included in final sample, with fitted line (N = 249,269). (DOCX 23 kb) [file 12916_2019_1279_MOESM5_ESM.docx]

**Additional file 5: Fig. S5**

**Scatter plot of mean age at marriage and proportion underweight by country of women age 20 to 49 included in final sample, with fitted line (N=249,269)**
