# Supplementary material for: Girl child marriage, socioeconomic status, and undernutrition: evidence from 35 countries in Sub-Saharan Africa
Source: BMC Med. 2019 Mar 8;17:55. doi: 10.1186/s12916-019-1279-8 (PMC6407221; doi:10.1186/s12916-019-1279-8)
Supplement: Supplementary file 6 — Table S6. Risk difference of girl child marriage (categorical specification) and underweight for pooled analysis (N = 249,269). Note. Coefficients presented are risk difference estimates from logistic regression models. 95% CIs in parentheses are based on cluster standard errors. Underweight is defined as body mass index less than 18.5. Model 1 adjusts for sampling cluster and woman’s primary education. Model 2 adjusts for woman’s primary education, woman’s age, age at first birth, number of children born, secondary education, wealth quintile, and partner characteristics. Bolded values are significant at the p < 0.05 level. ***p < 0.01, **p < 0.05 (DOCX 16 kb) [file 12916_2019_1279_MOESM6_ESM.docx]

**Additional file 6: Table S6**

| **Variables** | **Model 1** | | **Model 2** | |
| --- | --- | --- | --- | --- |
| Girl child marriage (18+ years, ref.) |  |  |  |  |
| Below 14 years | **-0·014***** | **(-0·024, -0·0039)** | -0·0071 | (-0·018, 0·0037) |
| 14-15 years | **-0·021***** | **(-0·0283, -0·013)** | **-0·016***** | **(-0·024, -0·0076)** |
| 16-17 years | **-0·022***** | **(-0·030, -0·015)** | **-0·021***** | **(-0·028, -0·013)** |
| Completion of primary education (no, ref.) | **-0·055***** | **(-0·064, -0·046)** | **-0·051***** | **(-0·060, -0·042)** |
| Current age (20-24 years, ref.) |  |  |  |  |
| 25-29 years |  |  | **-0·015***** | **(-0·024, -0·0069)** |
| 30-34 years |  |  | **-0·015***** | **(-0·024, -0·0049)** |
| 35-39 years |  |  | 0·0095 | (-0·010, 0·012) |
| 40-44 years |  |  | **0·013**** | **(0·00073, 0·025)** |
| 45-49 years |  |  | **0·033***** | **(0·020, 0·047)** |
| age at first birth (years) |  |  | -0·00042 | (-0·0014, 0·00054) |
| number of children ever born |  |  | **-0·0086***** | **(-0·010, -0·0072)** |
| Completion of secondary education (no, ref.) |  |  | **-0·080***** | **(-0·097, -0·062)** |
| Wealth quintile (poorest, ref.) |  |  |  |  |
| Poorer |  |  | **-0·014***** | **(-0·023, -0·0061)** |
| Middle |  |  | **-0·031***** | **(-0·039, -0·023)** |
| Richer |  |  | **-0·054***** | **(-0·064, -0·044)** |
| Richest |  |  | **-0·12***** | **(-0·14, -0·11)** |
| Age gap between partner and woman (years) |  |  | **-0·00045**** | **(-0·00082, -0·000081)** |
| Education gap between partner and woman (levels) |  |  | **-0·027***** | **(-0·033, -0·022)** |
|  |  |  |  |  |
| Coefficients presented are risk difference estimates from logistic regression models. 95% CIs in parentheses are based on cluster standard errors. Underweight is defined as body mass index less than 18·5. Model 1 adjusts for sampling cluster and woman's primary education. Model 2 adjusts for woman’s primary education, woman's age, age at first birth, number of children born, secondary education, wealth quintile, and partner characteristics. Bolded values are significant at the p<0·05 level. *** p<0·01, ** p<0·05 | | | | |
| Risk difference of girl child marriage (categorical specification) and underweight for pooled analysis (N=249,269) | | | | |
