# Supplementary material for: Girl child marriage, socioeconomic status, and undernutrition: evidence from 35 countries in Sub-Saharan Africa
Source: BMC Med. 2019 Mar 8;17:55. doi: 10.1186/s12916-019-1279-8 (PMC6407221; doi:10.1186/s12916-019-1279-8)
Supplement: Supplementary file 7 — Figure S7. Country-specific associations by girl child marriage category and underweight. (DOCX 25 kb) [file 12916_2019_1279_MOESM7_ESM.docx]

**Additional file 7: Fig. S7: Country-specific associations by girl child marriage category and underweight**

**Country-specific associations between girl child marriage (<14 years) and underweight conditional on full set of covariates**

Presents risk differences for marriage <14 years compared to 18+ years. All models control for primary education, age, age at first birth, number of children ever born, secondary education, wealth quintile, age gap, education gap, and EA fixed-effects. Based on 35 independent country-specific models.

**Country-specific associations between girl child marriage (14 to 15 years) and underweight conditional on full set of covariates**

Presents risk differences for marriage at 14 to 15 years compared to 18+ years. All models control for primary education, age, age at first birth, number of children ever born, secondary education, wealth quintile, age gap, education gap, and EA fixed-effects. Based on 35 independent country-specific models.

**Country-specific associations between girl child marriage (16 to 17 years) and underweight conditional on full set of covariates**

Presents risk differences for marriage at 16 to 17 years compared to 18+ years. All models control for primary education, age, age at first birth, number of children ever born, secondary education, wealth quintile, age gap, education gap, and EA fixed-effects. Based on 35 independent country-specific models.
