# Supplementary material for: Girl child marriage, socioeconomic status, and undernutrition: evidence from 35 countries in Sub-Saharan Africa
Source: BMC Med. 2019 Mar 8;17:55. doi: 10.1186/s12916-019-1279-8 (PMC6407221; doi:10.1186/s12916-019-1279-8)
Supplement: Supplementary file 8 — Table S8. Sample sizes for country-specific sensitivity analyses. (DOCX 14 kb) [file 12916_2019_1279_MOESM8_ESM.docx]

**Additional file 8: Table S8**

| Country | Sample Size |
| --- | --- |
| Benin | 14,887 |
| Burkina Faso | 11,261 |
| Burundi | 1,464 |
| Cameroon | 2,843 |
| Central African Republic | 1,018 |
| Chad | 10,011 |
| Comoros | 1,412 |
| Congo, Dem. Rep. | 5,335 |
| Congo, Rep. | 4,498 |
| Cote d'Ivoire | 945 |
| Ethiopia | 20,928 |
| Gabon | 1,398 |
| Gambia | 1,762 |
| Ghana | 4,237 |
| Guinea | 5,260 |
| Kenya | 10,953 |
| Lesotho | 1,009 |
| Liberia | 3,690 |
| Madagascar | 9,531 |
| Malawi | 12,362 |
| Mali | 18,879 |
| Mozambique | 9,391 |
| Namibia | 2,117 |
| Niger | 5,970 |
| Nigeria | 33,758 |
| Rwanda | 6,752 |
| Sao Tome and Principe | 867 |
| Senegal | 3,666 |
| Sierra Leone | 3,299 |
| Swaziland | 195 |
| Tanzania | 10,639 |
| Togo | 3,340 |
| Uganda | 3,913 |
| Zambia | 14,183 |
| Zimbabwe | 7,496 |

**Sample sizes for country-specific sensitivity analyses**
