# Supplementary material for: Girl child marriage, socioeconomic status, and undernutrition: evidence from 35 countries in Sub-Saharan Africa
Source: BMC Med. 2019 Mar 8;17:55. doi: 10.1186/s12916-019-1279-8 (PMC6407221; doi:10.1186/s12916-019-1279-8)
Supplement: Supplementary file 9 — Figure S9. Country-specific associations between girl child marriage (below 18 years) and severely underweight, conditional on full set of covariates. Note. All models control for primary education, age, age at first birth, number of children ever born, secondary education, wealth quintile, age gap, education gap, and EA fixed-effects. Based on 35 independent country-specific models. Two countries (Sao Tome and Principe and Swaziland) excluded due to lack of data or outcome variation by cluster. (DOCX 18 kb) [file 12916_2019_1279_MOESM9_ESM.docx]

**Additional file 9: Fig. S9**

**Country-specific associations between girl child marriage (below 18 years) and severely underweight, conditional on full set of covariates**

All models control for primary education, age, age at first birth, number of children ever born, secondary education, wealth quintile, age gap, education gap, and EA fixed-effects. Based on 35 independent country-specific models. Two countries (Sao Tome and Principe and Swaziland) excluded due to lack of data or outcome variation by cluster.
