# Supplementary material for: Girl child marriage, socioeconomic status, and undernutrition: evidence from 35 countries in Sub-Saharan Africa
Source: BMC Med. 2019 Mar 8;17:55. doi: 10.1186/s12916-019-1279-8 (PMC6407221; doi:10.1186/s12916-019-1279-8)
Supplement: Supplementary file 11 — Figure S11. Country-specific associations between girl child marriage (below 18 years) and underweight for women aged 20 to 24, conditional on full set of covariates. Note. All models control for primary education, age, age at first birth, number of children ever born, secondary education, wealth quintile, age gap, education gap, and EA fixed-effects. Based on 35 independent country-specific models. One country (Swaziland) excluded due to lack of data and outcome variation by cluster. (DOCX 22 kb) [file 12916_2019_1279_MOESM11_ESM.docx]

**Additional file 11: Fig S11**

0.0

**Country-specific associations between girl child marriage (below 18 years) and underweight for women aged 20 to 24, conditional on full set of covariates**

All models control for primary education, age, age at first birth, number of children ever born, secondary education, wealth quintile, age gap, education gap, and EA fixed-effects. Based on 35 independent country-specific models. One country (Swaziland) excluded due to lack of data and outcome variation by cluster.
