# Supplementary material for: Girl child marriage, socioeconomic status, and undernutrition: evidence from 35 countries in Sub-Saharan Africa
Source: BMC Med. 2019 Mar 8;17:55. doi: 10.1186/s12916-019-1279-8 (PMC6407221; doi:10.1186/s12916-019-1279-8)
Supplement: Supplementary file 12 — Figure S12. Country-specific associations between girl child marriage (below 18 years) and underweight for 2011 to 2014 data, conditional on full set of covariates. Note. All models control for primary education, age, age at first birth, number of children ever born, secondary education, wealth quintile, age gap, education gap, and EA fixed-effects. Based on 35 independent country-specific models. Ten countries excluded due to lack of data or outcome variation by cluster. (DOCX 18 kb) [file 12916_2019_1279_MOESM12_ESM.docx]

**Additional file 12: Fig. S12**

**Country-specific associations between girl child marriage (below 18 years) and underweight for 2011 to 2014 data, conditional on full set of covariates**

All models control for primary education, age, age at first birth, number of children ever born, secondary education, wealth quintile, age gap, education gap, and EA fixed-effects. Based on 35 independent country-specific models. Ten countries excluded due to lack of data or outcome variation by cluster.
