# Supplementary material for: Girl child marriage, socioeconomic status, and undernutrition: evidence from 35 countries in Sub-Saharan Africa
Source: BMC Med. 2019 Mar 8;17:55. doi: 10.1186/s12916-019-1279-8 (PMC6407221; doi:10.1186/s12916-019-1279-8)
Supplement: Supplementary file 13 — Figure S13. Country-specific associations between girl child marriage (below 18 years) and underweight controlling for work status of woman, conditional on full set of covariates. Note. All models control for primary education, age, age at first birth, number of children ever born, secondary education, wealth quintile, age gap, education gap, and EA fixed-effects. We additionally control for whether or not the woman worked in the past year. Based on 35 independent country-specific models. (DOCX 18 kb) [file 12916_2019_1279_MOESM13_ESM.docx]

**Additional file 13: Fig. S13**

**Country-specific associations between girl child marriage (below 18 years) and underweight controlling for work status of woman, conditional on full set of covariates**

All models control for primary education, age, age at first birth, number of children ever born, secondary education, wealth quintile, age gap, education gap, and EA fixed-effects. We additionally control for whether or not the woman worked in the past year. Based on 35 independent country-specific models.
