# Supplementary material for: Girl child marriage, socioeconomic status, and undernutrition: evidence from 35 countries in Sub-Saharan Africa
Source: BMC Med. 2019 Mar 8;17:55. doi: 10.1186/s12916-019-1279-8 (PMC6407221; doi:10.1186/s12916-019-1279-8)
Supplement: Supplementary file 14 — Table S14. Risk difference of girl child marriage (binary) and underweight for pooled analysis, excluding women married at age 18 or 19 (N = 184,828). Note. Coefficients presented are risk difference estimates from logistic regression models. Ninety-five percent CIs in parentheses are based on cluster standard errors. Underweight is defined as body mass index less than 18.5. Model 1 adjusts for sampling cluster and woman’s primary education. Model 2 adjusts for woman’s primary education, woman’s age, age at first birth, number of children born, secondary education, wealth quintile, and partner characteristics. Bolded values are significant at the p < 0.05 level. ***p < 0.01, **p < 0.05. (DOCX 16 kb) [file 12916_2019_1279_MOESM14_ESM.docx]

**Additional file 14: Table S14**

| **Variables** | **Model 1** | | **Model 2** | |
| --- | --- | --- | --- | --- |
|  |  |  |  |  |
| Girl child marriage (18+ years, ref.) | **-0·024***** | **(-0·032, -0·016)** | **-0·023***** | **(-0·031, -0·015)** |
| Completion of primary education (no, ref.) | **-0·056***** | **(-0·066, -0·046)** | **-0·051***** | **(-0·062, -0·041)** |
| Current age (20-24 years, ref.) |  |  |  |  |
| 25-29 years |  |  | **-0·012**** | **(-0·021, -0·0024)** |
| 30-34 years |  |  | **-0·014***** | **(-0·025, -0·0033)** |
| 35-39 years |  |  | 0·0037 | (-0·0084, 0·016) |
| 40-44 years |  |  | **0·014**** | **(0·00044, 0·027)** |
| 45-49 years |  |  | **0·034***** | **(0·019, 0·048)** |
| Age at first birth (years) |  |  | **-0·0010**** | **(-0·0020, -0·000056)** |
| Number of children ever born |  |  | **-0·0090***** | **(-0·011, -0·0074)** |
| Completion of secondary education (no, ref.) |  |  | **-0·080***** | **(-0·099, -0·060)** |
| Wealth quintile (poorest, ref.) |  |  |  |  |
| Poorer |  |  | **-0·016***** | **(-0·025, -0·0065)** |
| Middle |  |  | **-0·031***** | **(-0·040, -0·022)** |
| Richer |  |  | **-0·044***** | **(-0·055, -0·033)** |
| Richest |  |  | **-0·12***** | **(-0·14, -0·11)** |
| Age gap between partner and woman (years) |  |  | **-0·00026**** | **(-0·00066, 0·00014)** |
| Education gap between partner and woman (levels) |  |  | **-0·027***** | **(-0·034, -0·021)** |
|  |  |  |  |  |
| Coefficients presented are risk difference estimates from logistic regression models. 95% CIs in parentheses are based on cluster standard errors. Underweight is defined as body mass index less than 18·5. Model 1 adjusts for sampling cluster and woman's primary education. Model 2 adjusts for woman’s primary education, woman's age, age at first birth, number of children born, secondary education, wealth quintile, and partner characteristics. Bolded values are significant at the p<0·05 level. *** p<0·01, ** p<0·05 | | | | |
| **Risk difference of girl child marriage (binary) and underweight for pooled analysis, excluding women married at age 18 or 19 (N=184,828)** | | | | |
