# Supplementary material for: Girl child marriage, socioeconomic status, and undernutrition: evidence from 35 countries in Sub-Saharan Africa
Source: BMC Med. 2019 Mar 8;17:55. doi: 10.1186/s12916-019-1279-8 (PMC6407221; doi:10.1186/s12916-019-1279-8)
Supplement: Supplementary file 15 — Table S15. Regression results of unadjusted associations between girl child marriage and underweight by country. Note. Coefficients presented are from unadjusted linear regression models with 95% CIs in parentheses. Underweight is defined as body mass index less than 18.5. ***p < 0.01, **p < 005. (DOCX 17 kb) [file 12916_2019_1279_MOESM15_ESM.docx]

**Additional file 15: Table S15**

| Variable | Benin | Burkina Faso | Burundi | Cameroon | Central African Republic | Chad | Comoros | Congo , Dem. Rep. | Congo, Rep. | Cote d'Ivoire |
| --- | --- | --- | --- | --- | --- | --- | --- | --- | --- | --- |
|  |  |  |  |  |  |  |  |  |  |  |
| Married before age 18 (18+ years, ref.) | 1·010*** | 1·010 | 1·010 | 1·045*** | 0·994 | 1·031*** | 0·993 | 0·988 | 0·991 | 1·004 |
|  | (1·004 - 1·016) | (0·999 - 1·020) | (0·979 - 1·042) | (1·035 - 1·055) | (0·959 - 1·031) | (1·015 - 1·047) | (0·979 - 1·007) | (0·973 - 1·002) | (0·977 - 1·006) | (0·992 - 1·016) |
| Observations | 28,002 | 19,975 | 2,468 | 9,145 | 1,632 | 13,102 | 3,763 | 8,504 | 7,765 | 6,863 |

| Variable | Ethiopia | Gabon | Gambia | Ghana | Guinea | Kenya | Lesotho | Liberia | Madagascar | Malawi |
| --- | --- | --- | --- | --- | --- | --- | --- | --- | --- | --- |
|  |  |  |  |  |  |  |  |  |  |  |
| Married before age 18 (18+ years, ref.) | 1·027*** | 0·992 | 1·016 | 1·003 | 1·022*** | 1·022*** | 0·991 | 0·993 | 1·022*** | 0·994 |
|  | (1·014 - 1·039) | (0·980 - 1·004) | (0·991 - 1·042) | (0·993 - 1·012) | (1·007 - 1·037) | (1·014 - 1·030) | (0·982 - 1·000) | (0·981 - 1·005) | (1·007 - 1·036) | (0·987 - 1·001) |
| Observations | 22,875 | 4,928 | 2,745 | 12,766 | 8,541 | 24,597 | 6,364 | 7,192 | 12,658 | 23,015 |

| Variable | Mali | Mozambique | Namibia | Niger | Nigeria | Rwanda | Sao Tome and Principe | Senegal | Sierra Leone | Swaziland |
| --- | --- | --- | --- | --- | --- | --- | --- | --- | --- | --- |
|  |  |  |  |  |  |  |  |  |  |  |
| Married before age 18 (18+ years, ref.) | 1·014*** | 1·006 | 1·026*** | 1·042*** | 1·068*** | 0·995 | 0·994 | 1·008 | 1·013** | 0·994 |
|  | (1·006 - 1·022) | (0·999 - 1·013) | (1·007 - 1·046) | (1·026 - 1·059) | (1·062 - 1·073) | (0·985 - 1·004) | (0·973 - 1·016) | (0·994 - 1·024) | (1·000 - 1·026) | (0·983 - 1·004) |
| Observations | 24,896 | 19,053 | 6,764 | 12,322 | 46,800 | 15,812 | 1,595 | 8,693 | 7,303 | 2,193 |

| Variable | Tanzania | Togo | Uganda | Zambia | Zimbabwe |
| --- | --- | --- | --- | --- | --- |
|  |  |  |  |  |  |
| Married before age 18 (18+ years, ref.) | 0·994 | 1·005 | 1·003 | 0·994 | 0·995 |
|  | (0·986 - 1·002) | (0·991 - 1·020) | (0·991 - 1·015) | (0·987 - 1·001) | (0·988 - 1·003) |
| Observations | 19,657 | 6,115 | 10,244 | 24,633 | 16,073 |

Coefficients presented are from unadjusted linear regression models with 95% CIs in parentheses. Underweight is defined as Body Mass Index less than 18.5. *** p<0·01, ** p<0·05

**Regression results of unadjusted associations between girl child marriage and underweight by country**
